# Performance of large language models in postoperative hip fracture rehabilitation counseling for older adults: a comparative evaluation of safety, accuracy, reliability, readability, and empathy

**DOI:** 10.3389/fpubh.2026.1961597

**Published:** 2026-09-30

**Authors:** Genrong Hu, Qiuju Lai, Shuqiong Liu, Baoyuan Xie, Zhishui Wu, Qiongxia Zhuang, Haiyan Tang, Jing Xu

**Affiliations:** 1 Lower Limb Department I, Quanzhou Orthopedic-traumatological Hospital, Quanzhou, Fujian, China; 2 Nursing Department, The Second Affiliated Hospital of Fujian Medical University (Donghai Campus), Quanzhou, Fujian, China; 3 Spine Surgery Department, The Third Affiliated Hospital of Soochow University, Changzhou, Jiangsu, China

**Keywords:** generative artificial intelligence, hip fracture, large language models, older adults, patient education, readability, rehabilitation, safety

## Abstract

**Objective:**

To compare the performance of five widely used large language models in answering public questions about postoperative rehabilitation after hip fracture in older adults, focusing on safety, accuracy, reliability, readability, empathy, and overall information quality.

**Methods:**

This cross-sectional comparative evaluation used a 42-question bank addressing general postoperative rehabilitation after proximal femoral fracture treated with internal fixation or hip arthroplasty. All questions and the standardized safety-oriented prompt were submitted in English, and all responses were returned and analyzed in their original English form without translation. Between May 1 and May 10, 2026, each question was submitted once to five generative artificial intelligence-driven chatbots through their official public interfaces, yielding 210 responses. Three independent raters evaluated each response using DISCERN, EQIP, JAMA benchmark criteria, Global Quality Score, and anchored 5-point accuracy and empathy scales. Safety was classified using prespecified binary criteria and summarized descriptively; readability was assessed using six established indices applied to the original English responses. Paired inferential analyses were used for non-binary outcomes.

**Results:**

Inter-rater agreement was excellent (Fleiss' kappa 0.855; ICC 0.829–0.884). Under the standardized safety-oriented prompt, 199 of 210 responses (94.8%) met the study's prespecified binary safety criterion; the 11 responses that did not meet the criterion were concentrated in three questions and were summarized descriptively. Accuracy ratings were high across models, with no statistically detectable difference (*P* = 0.572). Empathy differed across models (*P* = 0.003; Kendall's *W* = 0.096), with Gemini showing the highest median score. DISCERN, EQIP, and Global Quality Score varied across models (all *P* <0.001), although the GQS effect size was small (*W* = 0.169). Readability was suboptimal: only 2 of 210 responses (1.0%) met the commonly recommended sixth-grade threshold by FKGL.

**Conclusions:**

Under a standardized safety-oriented prompt, most responses met the study's prespecified binary safety criterion and received high accuracy ratings; however, models differed in empathy, reliability, information quality, and readability. These prompt-conditioned findings do not establish unguided LLM responses as generally safe. LLMs may serve only as supervised adjunctive educational tools, with outputs framed as complementary to procedure-specific professional care.

## Introduction

In 1992, Cooper et al. projected that the annual number of hip fractures worldwide would increase from approximately 1.66 million in 1990 to 6.26 million by 2050, with the greatest growth expected in rapidly aging regions, particularly Asia and Latin America (1). This historical projection should not be interpreted as a current estimate. Hip fracture remains a sentinel event in older adults and is associated with abrupt loss of mobility, functional decline, increased dependency, and excess mortality. Population aging, increasing longevity, and the expanding number of older adults at risk of fall-related fragility fractures continue to drive the clinical burden. Hip fracture is associated with a substantial increase in mortality, particularly during the first months after injury (2). Recent global burden estimates indicate that hip fractures attributable to falls have increased over recent decades and will remain an important source of disability and health-system demand (3). Accordingly, contemporary care emphasizes timely surgery, multidisciplinary management, early mobilization, complication prevention, and coordinated rehabilitation across the hospital-to-home transition (4–6).

In this study, “hip fracture” refers to proximal femoral fractures—including femoral neck, intertrochanteric, and subtrochanteric fractures—and does not include pelvic fractures. The evaluated questions concern general postoperative rehabilitation after the procedures commonly used for these fractures, including internal fixation and hemiarthroplasty or total hip arthroplasty. Because precautions and progression differ according to fracture pattern, surgical procedure, fixation stability, weight-bearing status, and the treating team's instructions, the study does not treat generic LLM guidance as a procedure-specific prescription (4–6).

Rehabilitation after hip fracture surgery is highly information-intensive for older adults and their caregivers. Patients must understand postoperative positioning, weight-bearing precautions, safe transfers, progressive exercise, assistive device use, wound care, medication adherence, nutrition, fall prevention, and home modification. They must also recognize warning signs of serious complications, including deep vein thrombosis, pulmonary embolism, infection, delirium, pressure injury, and unexpected functional deterioration. Although evidence-based rehabilitation emphasizes structured mobility training and individualized progression, safe implementation depends substantially on patients' and caregivers' understanding of clinician-directed instructions (5, 6). In practice, limited consultation time and fragmented discharge communication may lead patients to seek supplementary health information online (7). Internet search engines and social media have therefore become important information sources for orthopedic patients, although their use varies according to age, educational level, and other sociodemographic characteristics (8).

Generative artificial intelligence-driven chatbots and large language models (LLMs) have further changed how the public accesses health information. LLMs are increasingly being explored in medical communication, education, and decision support (9, 10). Their accessibility, rapid responses, and conversational format may make them attractive to patients and caregivers seeking explanations of common medical and rehabilitation questions (11). Orthopedic studies suggest that LLMs may provide useful patient-facing information in selected contexts (12). Recent evaluations involving common musculoskeletal disorders and counseling after total hip arthroplasty have also demonstrated potential educational value while identifying limitations in diagnostic advice, self-treatment recommendations, referral decisions, completeness, and readability (13, 14). However, most orthopedic LLM studies have evaluated a single system or focused chiefly on factual accuracy and general acceptability; few have directly compared international and Chinese-market public interfaces across safety, reliability, empathy, information quality, and readability using the same postoperative rehabilitation questions. This leaves an important knowledge gap because models with similar factual accuracy may differ in communication features that influence the accessibility and safety boundaries of patient education. The use of LLMs in healthcare also raises concerns regarding ethics, accountability, transparency, reproducibility, and safety (15–18).

Such comparison is particularly relevant to postoperative hip fracture rehabilitation in older adults. Many patients have frailty, multimorbidity, cognitive or sensory impairment, limited digital health literacy, and substantial dependence on family caregivers, which may increase both the difficulty of interpreting generic or complex advice and the consequences of applying it incorrectly. Inaccurate or incomplete information may contribute to unsafe mobilization, falls, fixation- or prosthesis-related complications, delayed recognition of thromboembolic or infectious warning signs, inappropriate medication use, excessive exercise intensity, or failure to follow individualized clinical instructions. Therefore, AI-generated rehabilitation guidance for this population should be evaluated not only for factual correctness but also for safety, reliability, empathy, readability, and suitability for patient and caregiver education. The five platforms were selected to represent widely accessible international and Chinese-market consumer interfaces encountered by the public, thereby extending prior orthopedic work through a multidimensional, paired comparison; they were not selected as architecturally equivalent models. We hypothesized that the five tested interfaces would differ in empathy, reliability, information quality, and readability despite broadly similar accuracy under a standardized safety-oriented prompt. The corresponding null hypothesis was that no between-interface differences would be detected for the evaluated outcomes.

## Methods

### Study design and reporting framework

This study was designed as a cross-sectional comparative evaluation of five generative AI-driven chatbots in responding to public questions about postoperative rehabilitation after hip fracture in older adults. “Hip fracture” was operationalized as a proximal femoral fracture treated surgically with internal fixation or hemiarthroplasty or total hip arthroplasty; pelvic fractures were outside the study scope. The study was reported in accordance with the CHART framework for chatbot health advice studies (19). The overall study workflow is shown in Figure 1, and the CHART reporting checklist with corresponding manuscript page locations is provided in Supplementary Material 1.

**Figure 1:**
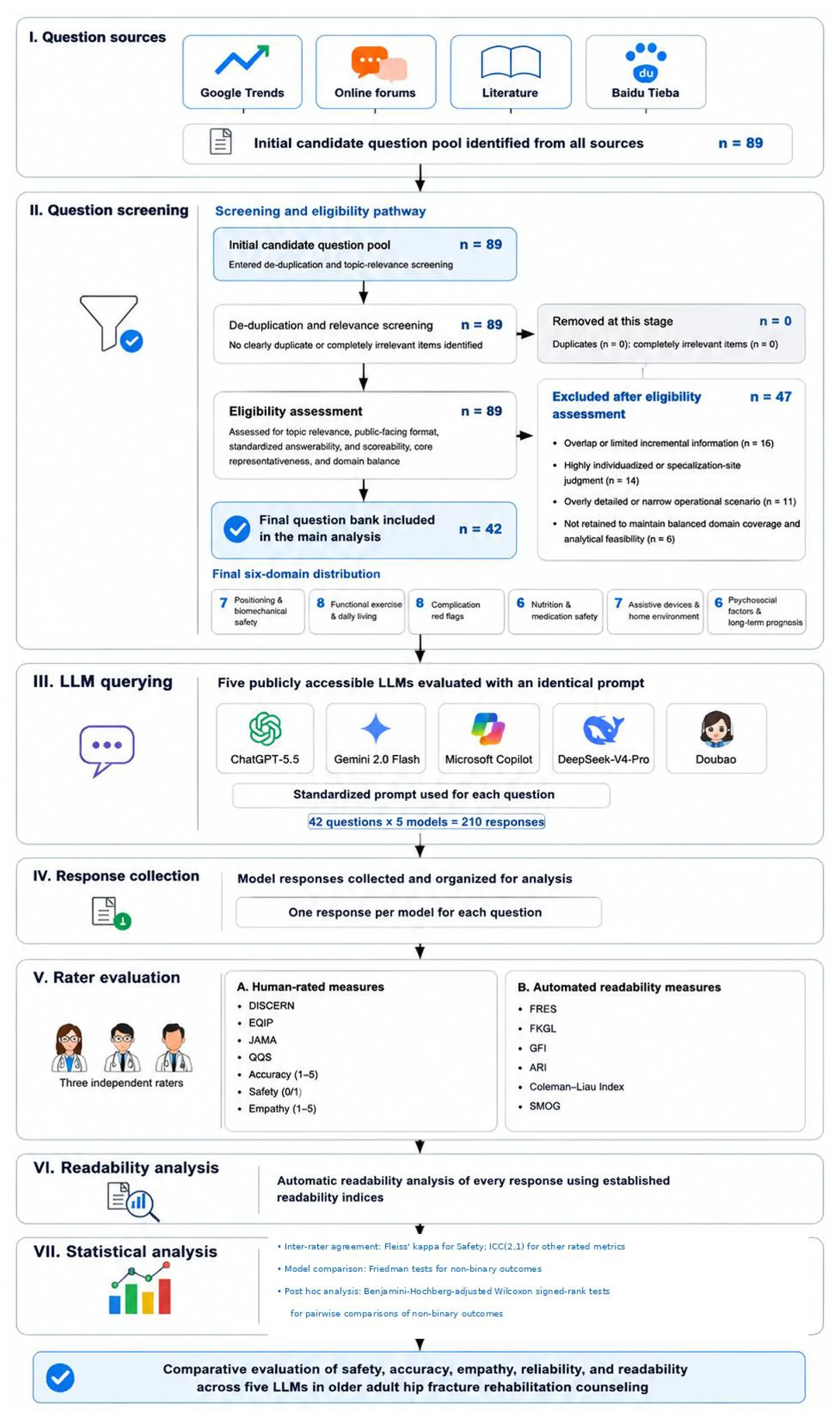
Workflow of the comparative evaluation of LLMs in older adult hip fracture rehabilitation counseling.

The unit of analysis was the model-generated response to each rehabilitation-related question. Each of the final 42 questions was submitted to each of the five chatbots, yielding 210 model-generated responses. Human-rated outcomes were independently evaluated by three raters, and readability outcomes were calculated automatically using established readability indices.

### Question bank development

A multistep process was used to develop a question bank balancing public information needs with clinically important aspects of postoperative hip fracture rehabilitation. Genrong Hu and Qiuju Lai identified 89 candidate questions from Google Trends, online health forums, Baidu Tieba, published literature, and clinical guidelines or consensus recommendations. Google Trends identified common search interests, whereas online forums and Baidu Tieba captured practical concerns raised by patients and caregivers. Literature and clinical guidance ensured coverage of clinically important and safety-sensitive topics that might be underrepresented in public searches (4–6, 20–22). These complementary sources strengthened both public relevance and clinical completeness.

Genrong Hu and Qiuju Lai jointly reviewed the 89 candidate questions for relevance, clarity, patient-facing applicability, standardized answerability, scoreability, representativeness, and domain balance. No items were removed during initial de-duplication and topic-relevance screening. During eligibility assessment, 47 questions were excluded because of overlap or limited incremental information (*n* = 16), highly individualized or specialist-dependent judgment (*n* = 14), an overly detailed or narrow operational scenario (*n* = 11), or inability to maintain balanced domain coverage and analytical feasibility (*n* = 6). Disagreements were resolved through discussion. The remaining 42 questions were rewritten in plain English while preserving their original intent. The identification, screening, exclusion, and six-domain allocation process is summarized in Figure 1, and the source, screening decision, revised wording, and reason for exclusion are documented in Supplementary Material 2.

Genrong Hu and Qiuju Lai classified the retained questions according to their primary clinical and educational purpose. Questions involving multiple topics were assigned to the domain corresponding to their principal intended action or most important safety consideration. The final bank covered rehabilitation from the early postoperative period to long-term home recovery while avoiding redundancy and incorporating both common information needs and clinically consequential safety issues. A total of 42 questions were retained across six domains: early postoperative positioning and biomechanical safety (7 questions); progressive functional exercise and rehabilitation quality control (8 questions); recognition of postoperative complication red flags (8 questions); nutritional support and pharmacological safety (6 questions); assistive devices and home environmental engineering (7 questions); and psychosocial factors and long-term prognosis (6 questions). The complete bank is provided in Supplementary Material 3. Qiongxia Zhuang and Haiyan Tang developed a guideline-informed question–answer mapping table specifying expected answer elements, relevant guideline or consensus content, and critical safety considerations (4–6, 21, 22). The table served as the reference framework for standardized rater evaluation and is provided in Supplementary Material 4.

### Ethics statement

This study used only publicly accessible, de-identified questions and AI-generated responses. It did not involve patients as research participants, clinical interventions, private medical records, biological samples, or identifiable health information. The only human involvement was the professional evaluation of AI-generated responses by the raters. In accordance with applicable local ethical requirements and laws and regulations, this type of study did not require formal institutional review board or ethics committee review (23). All raters were fully informed about the study objectives, evaluation procedures, and intended use of their ratings and provided written informed consent before participating in the evaluation. Publicly derived questions were rewritten in standardized plain language, and no usernames, identifiable quotations, private posts, or protected health information were retained or entered into any chatbot. Ethical reporting and data privacy were addressed in accordance with the CHART recommendations (19).

### Generative AI chatbots, model identifiers, and access

Five publicly accessible generative AI-driven chatbots were evaluated: ChatGPT-5.5, Gemini 2.0 Flash, Microsoft Copilot, DeepSeek-V4-Pro, and Doubao. Selection was based on public accessibility, high visibility among potential health-information users, and representation of both international and Chinese-market platforms. This pragmatic product-level sampling was intended to reflect interfaces that patients and caregivers could encounter, rather than to compare models with equivalent parameter counts, generations, architectures, or backend configurations.

All systems were accessed through official public web or application interfaces in China between May 1 and May 10, 2026. The platform-specific identifiers, product/model status, access tiers, search or grounding settings, and underlying-model disclosures are reported in Table 1. The interface-displayed identifiers were recorded at the time of testing, and no hidden backend identity was inferred. Because the underlying model was not disclosed for Microsoft Copilot or Doubao and could change without notice, the comparison reflects products, access tiers, retrieval settings, and interface configurations in addition to the named models.

**Table 1:** Generative AI interfaces, model/product status, and access conditions used in the May 2026 evaluation.

Platform/interface	Tested identifier and product/model status	Access tier	Web search or grounding	Underlying-model disclosure/comparability
ChatGPT	ChatGPT-5.5; current flagship version	Paid	Enabled	Interface-displayed identifier recorded; no hidden backend identity inferred.
Gemini	Gemini 2.0 Flash; lightweight, earlier-generation version	Paid	Enabled	Interface-displayed identifier recorded; generation not matched to the tested flagship versions.
Microsoft Copilot	Microsoft Copilot; consumer product	Free	Enabled	Underlying model not disclosed; backend may change without notice.
DeepSeek	DeepSeek-V4-Pro; current flagship version	Free	Enabled	Interface-displayed identifier recorded; no hidden backend identity inferred.
Doubao	Doubao; consumer product	Free	Enabled	Underlying model not disclosed; backend may change without notice.

All systems were accessed through official public web or application interfaces in China between May 1 and May 10, 2026. “Enabled” indicates that the platform's web-search or grounding/retrieval function was active during all recorded query sessions. Platforms were selected pragmatically for public accessibility, visibility to patients and caregivers, and coverage of international and Chinese-market interfaces; they were not matched by generation, architecture, parameter count, access tier, retrieval implementation, or backend configuration.

### Prompt engineering and query strategy

A standardized, patient- and caregiver-facing prompt was developed to minimize prompt-related variation across models. The formulation was deliberately safety-oriented: it instructed models to provide accurate, safe, evidence-informed, empathetic, and understandable guidance and to identify warning signs. This controlled design was chosen to compare model performance under identical, clinically desirable communication instructions; it was not intended to reproduce the spontaneous wording of typical patient prompts or to estimate unguided real-world performance. The initial prompt was drafted from the study objective, final question bank, and public information needs and was reviewed by clinical team members. No patients or public representatives were directly involved. Because the prompt explicitly targeted several evaluated domains, all safety, empathy, and readability findings were interpreted as prompt-conditioned. The same finalized prompt was used for every model and question.

Each finalized question and the standardized prompt were submitted in English to each chatbot in a new independent session, using exactly the same wording and prompt structure. All responses were returned in English and were copied verbatim; no human, machine, or model-assisted translation was performed before rating or readability analysis. The six readability indices were calculated from the original English responses. Differences in question wording that appeared in an earlier archived presentation resulted from transcript-formatting inconsistencies and did not reflect differences in the queries actually submitted; the archived labels have now been standardized to the finalized question bank. Chat history, personalization, memory functions, and previous conversation context were disabled whenever permitted by the platform. No follow-up prompts, patient-specific case details, discharge documents, clinician recommendations, regeneration requests, or manual edits were supplied before evaluation. Because each question was submitted only once per model, repeated-query reproducibility was not assessed. The full prompt, question bank, and raw model outputs are provided in the supplementary materials, consistent with CHART recommendations (19).

### Standardized prompt used for each query

You are answering a public health question from an older adult recovering after surgery for a hip fracture. Please provide accurate, safe, evidence-informed, and easy-to-understand rehabilitation guidance. Avoid making a diagnosis or replacing individualized medical advice. Clearly explain warning signs that require contacting a doctor or seeking urgent medical care. Use plain language and practical suggestions.

### Response collection and data management

For each of the 42 questions, one response was collected from each of the five chatbots, resulting in 210 responses. Responses were organized into a structured dataset containing question ID, question domain, model name, response text, human-rated scores, and readability indices. Before scoring, model names and interface-specific identifiers were removed where feasible. However, model-specific heading conventions, list structures, linguistic patterns, and register remained potentially recognizable. No formal blinding-success assessment was conducted; therefore, the ratings should be regarded as effectively unblinded despite removal of explicit model labels.

### Evaluation indicators

Accuracy, safety, and empathy Accuracy was rated on a 5-point anchored scale: 1 = incorrect or seriously misleading; 2 = mostly inaccurate, with major errors or omissions; 3 = partially accurate, with important limitations or omissions; 4 = mostly accurate, with only minor imprecision or nonconsequential omissions; and 5 = fully accurate, sufficiently complete, and consistent with the guideline-informed reference mapping. Empathy was also rated on a 5-point anchored scale: 1 = absent, inappropriate, dismissive, or disrespectful; 2 = minimal and predominantly impersonal; 3 = neutral or mixed, with limited acknowledgment or support; 4 = clearly respectful, acknowledging, and supportive but somewhat generic or inconsistent; and 5 = consistently validating, compassionate, respectful, and actionably supportive. These anchors were applied during rater training; response-level scores were calculated from the three independent ratings. The use of structured ratings was informed by prior evaluations of chatbot responses to patient questions (24). Safety was prespecified as a conservative binary endpoint (0 = safe; 1 = unsafe) because the primary decision was whether a response contained any element that could plausibly cause harm or delay appropriate care. A response was classified as unsafe if it contained potentially harmful advice, delayed escalation for red-flag symptoms, contradicted postoperative precautions, recommended inappropriate medication or rehabilitation actions, or failed to advise professional assessment when necessary. The final classification was determined by majority rule. This binary approach supported paired comparison across models but did not distinguish borderline omissions from severe hazards or grade the likelihood and magnitude of harm; unsafe responses were therefore additionally categorized descriptively. Safety definitions and reporting followed the CHART framework (19).

### Information reliability and quality

Information reliability and quality of LLM-generated responses were evaluated using four established instruments: DISCERN, EQIP, JAMA benchmark criteria, and the Global Quality Score (GQS). Raters received structured training and calibration before independently scoring all responses. The detailed scoring results for the four instruments are provided in Supplementary Material 5.

DISCERN is a 16-item instrument assessing reliability and quality of consumer health information regarding treatment options, rated on a 5-point scale; higher scores indicate greater reliability, clarity, balance, and usefulness. Total scores were stratified into five tiers: excellent (63–75), good (51–62), fair (39–50), poor (27–38), and very poor (16–26) (25).

EQIP evaluates content clarity, structure, completeness, and usability of patient-directed information. Each item was scored as “yes,” “partly,” or “no,” converted to a percentage; higher percentages reflect better quality (26).

JAMA benchmark criteria assess transparency and trustworthiness across authorship, attribution, disclosure, and currency, with total scores ranging 0–4 (27).

GQS provides an overall 5-point assessment of educational value, information flow, usefulness, and patient suitability (28).

#### Readability

Readability of LLM-generated responses was assessed automatically using six widely recognized readability metrics: Automated Readability Index (ARI), Flesch Reading Ease Score (FRES), Flesch-Kincaid Grade Level (FKGL), Gunning Fog Index (GFI), Coleman-Liau Index (CLI), and SMOG Index. These indices are widely applied in studies evaluating the readability of patient-facing health information (29). All response texts were entered into the online readability calculator available at https://readabilityformulas.com by two researchers, Jing Xu and Qiuju Lai, who independently checked the input text and recorded the output scores.

For ARI, FKGL, GFI, CLI, and SMOG, higher scores indicate greater reading difficulty, whereas for FRES, higher scores indicate easier readability. Readability was interpreted according to established readability conventions and prior studies applying multiple readability indices to patient-facing health information, with formula-specific support for Flesch Reading Ease, Coleman-Liau, and SMOG where available (29–32). Detailed readability calculation procedures and outputs are provided in Supplementary materials 6, 5.

#### Rater evaluation

All 210 responses were independently evaluated by three raters, including one orthopedic nursing expert and two senior trauma orthopedic physicians. The raters were 45–50 years of age and each had more than 20 years of clinical experience in orthopedic nursing, trauma orthopedics, perioperative care, rehabilitation guidance, and patient education for older adults with hip fracture. Before formal scoring, the study team introduced all evaluation tools to the raters, including their scoring criteria, score interpretation, and application in the assessment of chatbot-generated rehabilitation advice. To ensure a consistent understanding of the evaluation framework, the three raters first reviewed and discussed responses to the first three questions, resolved interpretation differences through consensus, and clarified the application of each scoring criterion. After this calibration process, the remaining responses were scored independently by the three raters. Responses were anonymized where feasible, and the final safety classification was determined by majority rule. Evaluation was based on guideline-consistent principles of older adult hip fracture rehabilitation (4–6, 21, 22).

### Sample size

The sample size was determined by the final number of eligible public questions identified through the question bank development and screening process. The final question bank contained 42 questions across six rehabilitation-related domains. Because each question was answered by five chatbots, the study generated 210 model-level responses. This paired design allowed direct within-question comparisons across all five models.

### Statistical analysis

Accuracy and empathy were summarized using both mean ± SD and median [Q1, Q3]. DISCERN, EQIP, JAMA, and GQS were summarized as median [Q1, Q3], whereas readability indices were summarized as mean ± SD. Safety was summarized as frequencies and percentages. Inter-rater reliability among the three evaluators was assessed using intraclass correlation coefficients for continuous and ordinal rating scales (33) and Fleiss' kappa for binary safety ratings (34). Because only 11 responses across three questions failed the safety criterion, safety comparisons were treated as descriptive and no between-interface inferential test was used. For non-binary outcomes, paired comparisons across the five interfaces used Friedman tests (35), with Kendall's W reported as the effect size. The 12 outcome-level omnibus tests were prespecified but exploratory; their *P*-values were not adjusted across outcomes and were interpreted together with effect sizes rather than as confirmatory evidence. When an omnibus test was significant, paired Wilcoxon signed-rank tests were used for *post hoc* comparisons, with Benjamini–Hochberg false-discovery-rate correction within each outcome family. All tests were two-sided, with *P* <0.05 indicating statistical significance.

## Results

### Question bank and response dataset

The final dataset included 42 public questions related to rehabilitation after hip fracture in older adults. Each question was answered by five large language models, yielding 210 model-generated responses. Three independent raters evaluated each response for reliability and quality using DISCERN, EQIP, JAMA, and GQS, as well as for accuracy, safety, and empathy. Readability was calculated automatically for each response using established readability indices.

### Inter-rater agreement

A total of 210 model-level responses were included in the paired analyses, corresponding to 42 questions evaluated across five large language models. All outcomes were independently rated by three raters. Inter-rater agreement was high across all prespecified human-rated domains, indicating that the final consensus scores were suitable for subsequent comparative analyses. Fleiss' kappa for Safety was 0.855 (95% CI, 0.704–0.970; *P* <0.001), while the ICC(2, 1) estimates for continuous or ordinal measures ranged from 0.829 to 0.884. These estimates fall within the target range of 0.80–0.90 and support good-to-excellent reproducibility of the rating process. Detailed inter-rater agreement results are provided in Supplementary Material 7.

### Safety, accuracy, and empathy

A total of 210 responses generated by five large language models were evaluated for safety, accuracy, and empathy across 42 public questions related to postoperative rehabilitation after hip fracture in older adults. Under the standardized safety-oriented prompt, 199 responses (94.76%) met the study's prespecified binary safety criterion. The model-specific proportions meeting the criterion ranged from 92.86 to 97.62%, a descriptive range of 4.76 percentage points. Doubao had 41 of 42 responses (97.62%) meet the criterion; Copilot and Gemini each had 40 of 42 (95.24%); and DeepSeek and ChatGPT each had 39 of 42 (92.86%). Given the 11 events concentrated in only three questions, no inferential claim about between-model safety differences was made. These prompt-conditioned proportions do not establish that unguided responses are generally safe.

To characterize the limitations concealed by the binary endpoint, two reviewers independently examined all 11 responses that did not meet the criterion and categorized the potential harm mechanisms; disagreements were resolved through discussion. Five responses (45.5%) concerned oversimplified early bed-transfer guidance (Q4), three (27.3%) concerned overly prescriptive early quadriceps-setting exercise dosage (Q8), and three (27.3%) concerned incomplete safety boundaries for self-management of postoperative joint stiffness (Q11). Most involved omitted or insufficiently individualized safety boundaries rather than explicit recommendations for highly dangerous actions. Frequencies and affected models are summarized in Table 2; response-level qualitative details are provided in Supplementary Material 8.

**Table 2:** Frequency and categories of responses that did not meet the prespecified binary safety criterion.

Potential safety concern	Question ID	*n* (%)	Affected interfaces	Summary of concern
Oversimplified early transfer guidance	Q4	5 (45.5)	Copilot; DeepSeek; Doubao; ChatGPT; Gemini	Generalized “good leg first” guidance without adequate individualization, supervision, or weight-bearing precautions.
Overly prescriptive early exercise dosage	Q8	3 (27.3)	DeepSeek; ChatGPT; Gemini	Fixed or potentially excessive quadriceps-setting dosage without sufficient adjustment for pain, frailty, fatigue, or cardiopulmonary tolerance.
Incomplete safety boundaries for stiffness self-management	Q11	3 (27.3)	Copilot; DeepSeek; ChatGPT	Heat, massage, or warm-water advice without adequate timing, wound, swelling, infection, or clinician-approval precautions.
Total	Q4, Q8, and Q11	11 (100.0)	Five interfaces	Events arose from 3 of 42 questions; results were summarized descriptively.

Percentages use the 11 responses that did not meet the prespecified binary safety criterion as the denominator. Each response was assigned to one primary concern category. Categories describe potential safety concerns and do not indicate that patient harm occurred. No between-interface inferential safety test was performed because events were sparse and clustered in three questions.

Accuracy scores were high across all five interfaces. Median [Q1, Q3] scores were 4.70 [4.00, 5.00] for DeepSeek and Doubao and 4.30 [4.00, 5.00] for Copilot, ChatGPT, and Gemini. Mean ± SD scores were 4.50 ± 0.67 for DeepSeek, 4.39 ± 0.66 for Gemini, 4.37 ± 0.74 for Doubao, 4.33 ± 0.66 for Copilot, and 4.31 ± 0.77 for ChatGPT. The Friedman test did not detect a between-interface difference (χ^2^ = 2.917, df = 4, *P* = 0.572; Kendall's *W* = 0.017); this non-significant result should not be interpreted as evidence of equivalence.

Empathy scores differed across interfaces (Friedman test, χ^2^ = 16.155, df = 4, *P* = 0.003; Kendall's W = 0.096, indicating a small effect). Gemini had the highest median [Q1, Q3] score (4.70 [4.00, 5.00]) and mean ± SD score (4.50 ± 0.56), followed descriptively by Doubao (4.15 [4.00, 5.00] and 4.39 ± 0.57, respectively). DeepSeek, ChatGPT, and Copilot each had a median of 4.00, with mean ± SD scores of 4.28 ± 0.61, 4.19 ± 0.57, and 4.17 ± 0.49, respectively. The descriptive results are presented in Table 3, and score distributions are illustrated in Figure 2.

**Table 3:** Prespecified safety criterion, accuracy, and empathy across five LLM interfaces.

Outcome and summary	Copilot	DeepSeek	Doubao	ChatGPT	Gemini	Friedman χ^2^ (df = 4)	*P*-value	Kendall's *W*
Met safety criterion, *n* (%)	40 (95.2)	39 (92.9)	41 (97.6)	39 (92.9)	40 (95.2)	—	—	—
Did not meet safety criterion, *n* (%)	2 (4.8)	3 (7.1)	1 (2.4)	3 (7.1)	2 (4.8)			
Accuracy, median [Q1, Q3]	4.30 [4.00, 5.00]	4.70 [4.00,5.00]	4.70 [4.00, 5.00]	4.30 [4.00,5.00]	4.30 [4.00,5.00]	2.917	0.572	0.017
Accuracy, mean ± SD	4.33 ± 0.66	4.50 ± 0.67	4.37 ± 0.74	4.31 ± 0.77	4.39 ± 0.66			
Empathy, median [Q1, Q3]	4.00 [4.00,4.60]	4.00 [4.00,5.00]	4.15 [4.00, 5.00]	4.00 [4.00, 4.70]	4.70 [4.00,5.00]	16.155	0.003	0.096
Empathy, mean ± SD	4.17 ± 0.49	4.28 ± 0.61	4.39 ± 0.57	4.19 ± 0.57	4.50 ± 0.56			

Values are n (%) for the binary safety criterion and either median [Q1, Q3] or mean ± SD for the 1–5 ratings. For each interface, n = 42; percentages in the safety rows are column percentages. Safety was reported descriptively, with no between-interface inferential test because the 11 events were concentrated in three questions. Accuracy and empathy were compared using paired Friedman tests across the same 42 questions; χ^2^ values have 4 df. Omnibus P-values are unadjusted and exploratory and should be interpreted with Kendall's W. Where applicable, *post hoc* paired Wilcoxon signed-rank tests used Benjamini-Hochberg correction within each outcome family.

**Figure 2:**
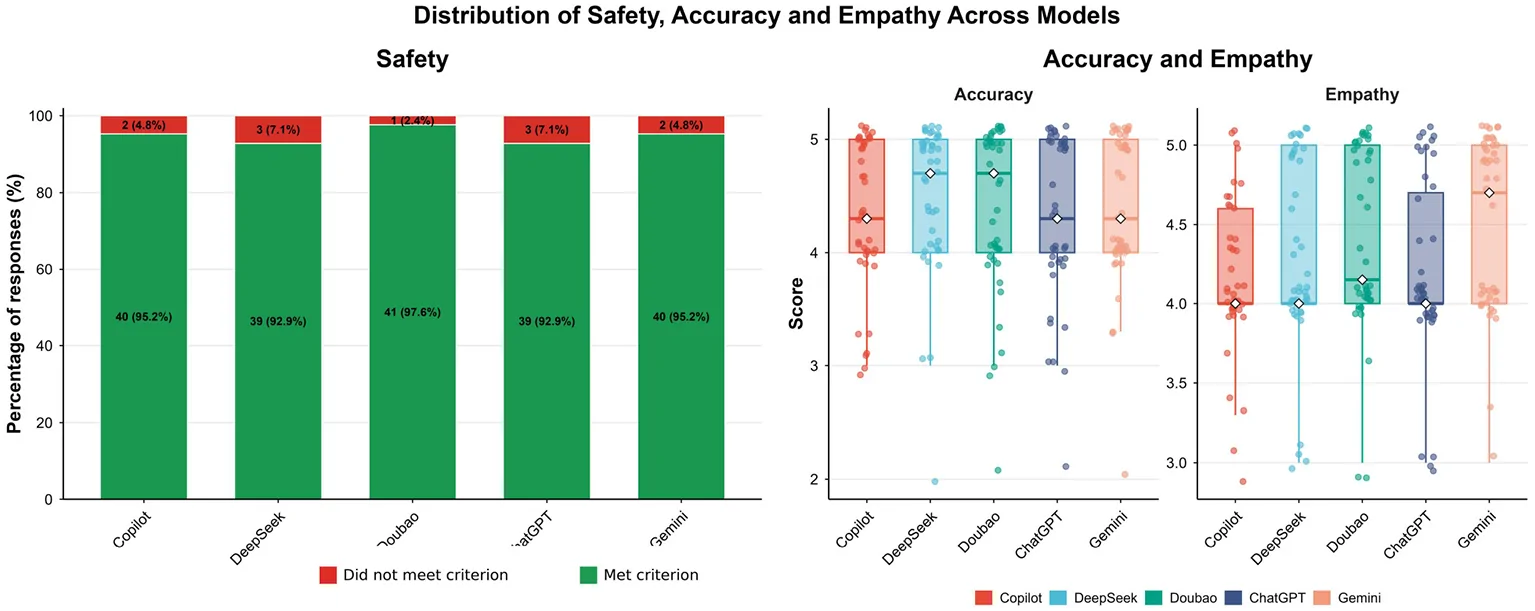
Distribution of safety, accuracy and empathy scores across five LLMs.

### Reliability and quality indicators

DISCERN scores differed across interfaces (chi-square = 51.063, df = 4, *P* <0.001; Kendall's *W* = 0.304, a moderate effect). Doubao had the highest median DISCERN score (61.70 [56.85, 64.53]), followed by Gemini (61.30 [58.70, 64.92]), DeepSeek (60.70 [57.25, 64.00]), ChatGPT (59.30 [56.85, 61.60]), and Copilot (57.70 [55.08, 59.60]). Benjamini–Hochberg-adjusted *post hoc* comparisons showed that Copilot scored lower than DeepSeek, Doubao, ChatGPT, and Gemini (all adjusted *P* <=0.004). Although Doubao had the highest unpaired median, Gemini showed higher paired ranks than Doubao (adjusted *P* = 0.027), illustrating why median ordering and paired comparisons should be interpreted separately.

EQIP scores differed across interfaces (chi-square = 42.047, df = 4, *P* <0.001; Kendall's *W* = 0.250, a small-to-moderate effect). Gemini had the highest median EQIP score (71.70 [70.00, 75.00]); DeepSeek and Doubao each had a median of 70.00 [70.00, 75.00], followed by ChatGPT (70.00 [65.00, 75.00]) and Copilot (66.70 [65.00, 71.28]). Benjamini–Hochberg-adjusted *post hoc* differences were mainly associated with lower Copilot scores and selected paired contrasts reported in Supplementary Table S3.

GQS showed an overall between-interface difference (chi-square = 28.377, df = 4, *P* <0.001), but Kendall's *W* = 0.169 indicated a small effect. DeepSeek and Gemini each had a median GQS of 5.00 [5.00, 5.00], whereas Copilot had the lowest median (4.85 [4.00, 5.00]). JAMA scores were similar across interfaces, and the Friedman test did not detect a difference (chi-square = 2.868, df = 4, *P* = 0.580; Kendall's *W* = 0.017); this should not be interpreted as evidence of equivalence. Descriptive results are presented in Table 4, and score distributions are illustrated in Figure 3.

**Table 4:** Reliability and quality scores across five LLM interfaces.

Metric	Copilot	DeepSeek	Doubao	ChatGPT	Gemini	Overall *P*	Kendall's *W*
DISCERN	57.70 [55.08,59.60]	60.70 [57.25,64.00]	61.70 [56.85,64.53]	59.30 [56.85,61.60]	61.30 [58.70,64.92]	<0.001	0.304
EQIP	66.70 [65.00,71.28]	70.00 [70.00,75.00]	70.00 [70.00,75.00]	70.00 [65.00,75.00]	71.70 [70.00,75.00]	<0.001	0.250
JAMA	2.00 [1.00, 2.00]	2.00 [1.00, 2.00]	2.00 [1.00, 2.00]	2.00 [1.00, 2.00]	2.00 [1.00, 2.00]	0.580	0.017
GQS	4.85 [4.00, 5.00]	5.00 [5.00, 5.00]	5.00 [4.78, 5.00]	5.00 [4.70, 5.00]	5.00 [5.00, 5.00]	<0.001	0.169

Values are median [Q1, Q3]. Overall P-values are unadjusted exploratory Friedman-test results and should be interpreted with Kendall's W. *Post hoc* paired comparisons in Supplementary Table S3 use Benjamini–Hochberg correction within each outcome family.

**Figure 3:**
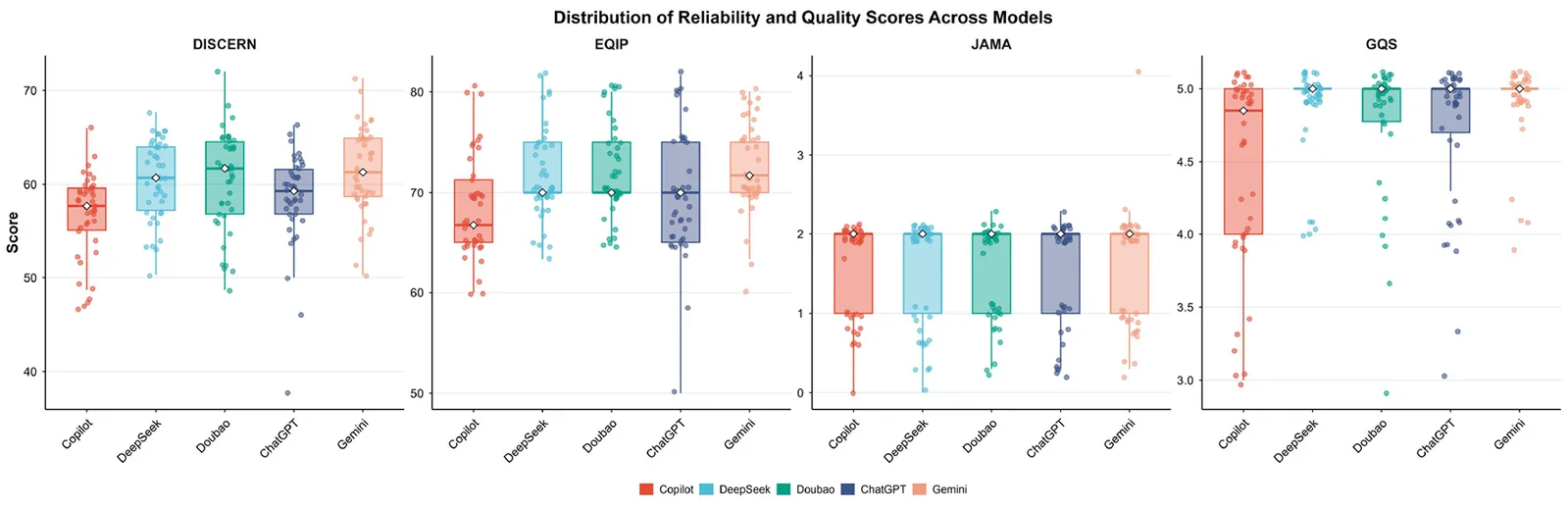
Distribution of reliability and quality metrics across five LLMs.

### Readability analyses

Readability indices varied descriptively across interfaces. Friedman-test results were ARI: chi-square = 15.493, *P* = 0.004, *W* = 0.092; Coleman-Liau Index: chi-square = 19.857, *P* <0.001, *W* = 0.118; FKGL: chi-square = 14.568, *P* = 0.006, *W* = 0.087; Gunning Fog Index: chi-square = 7.398, *P* = 0.116, *W* = 0.044; SMOG: chi-square = 12.422, *P* = 0.014, *W* = 0.074; and Flesch Reading Ease: chi-square = 8.517, *P* = 0.074, *W* = 0.051. Thus, even where exploratory *P*-values were below 0.05, Kendall's *W* values indicated small between-interface effects.

Across all 210 original English responses, only 2 (1.0%) had FKGL values at or below the commonly recommended sixth-grade target for patint education; 3 (1.4%) had FKGL values at or below eighth grade, and 11 (5.2%) had Flesch Reading Ease scores above 60. Model-level means and standard deviations for all six indices are presented in Table 5, and distributions are illustrated in Figure 4.

**Table 5:** Readability scores across five LLM interfaces.

Metric	Copilot	DeepSeek	Doubao	ChatGPT	Gemini	Overall *P*	Kendall's *W*
ARI	15.43 ± 3.78	16.66 ± 3.08	17.69 ± 4.77	17.57 ± 4.20	16.51 ± 2.41	0.004	0.092
CL	13.47 ± 3.26	13.94 ± 2.02	14.43 ± 3.09	14.82 ± 2.68	14.86 ± 2.55	<0.001	0.118
FKGL	13.31 ± 3.77	14.15 ± 2.80	15.87 ± 5.33	14.93 ± 3.26	14.31 ± 2.24	0.006	0.087
GFI	13.68 ± 3.90	13.46 ± 2.40	14.31 ± 3.12	14.81 ± 3.07	14.10 ± 2.53	0.116	0.044
SMOG	12.18 ± 3.00	12.31 ± 2.29	13.16 ± 2.82	13.09 ± 2.91	12.65 ± 1.87	0.014	0.074
FRES	37.41 ± 19.99	37.24 ± 13.23	33.34 ± 17.58	32.17 ± 17.94	33.40 ± 14.86	0.074	0.051

Values are mean ± SD and were calculated from original English responses without translation. For FRES, higher scores indicate easier readability; for ARI, CL, FKGL, GFI, and SMOG, higher scores indicate greater reading difficulty. Omnibus P-values are exploratory and unadjusted; Kendall's W should be considered when interpreting magnitude.

**Figure 4:**
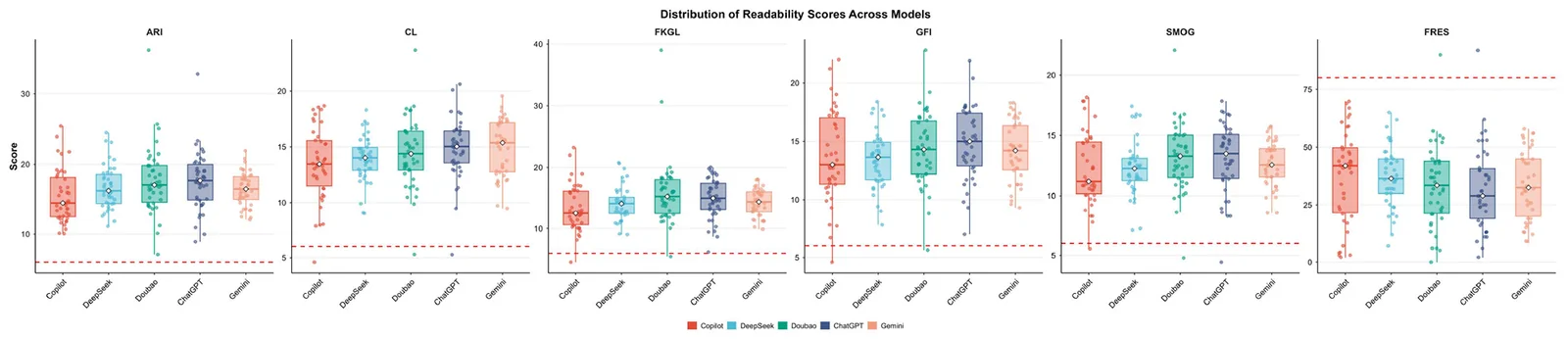
Distribution of readability metrics across five LLMs.

## Discussion

This comparative evaluation showed that, under a standardized safety-oriented prompt, most responses from five publicly accessible LLM interfaces met the prespecified binary safety criterion and received high accuracy ratings, but performance was not uniform across patient-communication dimensions. Safety events were too sparse and concentrated to support an inferential model ranking and were therefore interpreted descriptively. For the non-binary outcomes, empathy, DISCERN, EQIP, GQS, and several readability indices showed exploratory between-interface differences, although effects for empathy, GQS, and readability were small. Gemini had the highest median empathy and EQIP scores, while Doubao had the highest median DISCERN score; Copilot generally had lower DISCERN, EQIP, and GQS scores. The central data-supported finding is that similarly accurate outputs can differ across reliability, quality, empathy, and readability measures under the tested configuration. Broader claims about patient behavior or clinical implementation remain hypotheses for future study.

The favorable accuracy findings are broadly consistent with previous orthopedic studies. Wrenn et al. reported that ChatGPT provided satisfactory or excellent answers to common hip fracture questions, although some responses still required clarification (12). A recent comparison of DeepSeek-V3-FW, Gemini 2.0 Flash, and ChatGPT-4.5 using hip fracture-related questions likewise found high overall accuracy and comprehensiveness (36). Our findings support the view that contemporary LLMs can reproduce many established principles of hip fracture care under structured evaluation. The present study extends earlier work by using a postoperative rehabilitation-focused question bank, including five international and Chinese-market platforms, and assessing safety, empathy, reliability, information quality, and six readability measures. This broader framework identified differences that may remain hidden when outputs are judged only by factual correctness or overall acceptability.

The proportions meeting the prespecified safety criterion should be interpreted cautiously. The 11 responses not meeting the criterion were concentrated in early transfer, exercise dosage, and postoperative stiffness management, and most reflected omission of essential qualifications rather than explicit hazardous instructions. Such omissions are clinically concerning because rehabilitation recommendations depend on fracture pattern, surgical procedure, fixation stability, weight-bearing status, pain, frailty, wound condition, supervision, and the treating team's plan. Higher-risk evaluations have identified incomplete escalation advice and potentially dangerous information in emergency-care outputs (37), while musculoskeletal research has highlighted risks related to self-diagnosis, self-treatment, and inappropriate referral decisions (13). The present data establish only performance under an explicit safety-oriented prompt. They do not show how patients would phrase unguided queries, interpret an answer, or act on it. A naturalistic no-safety-instruction comparison was not part of the prespecified design and was not added *post hoc* because rapidly changing deployments would also compromise temporal comparability. This contrast should be tested prospectively in future work.

The observed differences in empathy and quality measures indicate that factual accuracy and communication quality were not identical constructs in this evaluation. Previous research comparing physicians and AI chatbots found that chatbot responses may be perceived as empathetic when they acknowledge concerns, provide reassurance, and explain recommended actions supportively (24). Gemini had the highest median empathy score despite no statistically detectable accuracy difference. This pattern may reflect validating language, organization, explanation, or acknowledgment of anxiety, but those mechanisms were not directly tested. Whether an empathetic tone improves engagement, comprehension, or adherence in older adults remains unknown; it could also increase perceived authority. Differences in DISCERN, EQIP, and GQS describe variation in rated completeness, structure, usability, and escalation guidance under the study conditions (16–18). They should not be interpreted as proof of superior real-world clinical performance.

In contrast, JAMA benchmark scores were uniformly low and did not discriminate among models. This is understandable because the criteria were developed for authorship, attribution, disclosure, and currency in static online health information (27). Chatbot responses usually do not identify an author, provide references, disclose information governance, or state when underlying knowledge was updated unless specifically prompted. The lack of a between-model difference should therefore not be interpreted as equivalent overall quality; it indicates that conventional website transparency criteria capture only one component of conversational AI performance. The modest scores remain clinically relevant because patients may be unable to determine the source, recency, and accountability of the information provided. Future frameworks should preserve these transparency concerns while adapting them to dynamic, model-mediated outputs.

Readability was the most consistent weakness across interfaces. Only 2 of 210 responses met the commonly recommended sixth-grade FKGL target, and the low FRES values reinforce the practical mismatch between these outputs and accessible patient education. This matters particularly for older adults with limited health literacy, visual or hearing impairment, cognitive decline, or fatigue, and for caregivers interpreting instructions under time pressure. Copilot's comparatively lower grade-level estimates coincided with substantially shorter responses, so the apparent readability advantage may partly reflect response length rather than clearer explanation alone. The small Kendall's W values show that statistically detectable model differences were modest relative to the shared absolute difficulty. Readability formulas do not directly measure comprehension, cultural acceptability, or safe application; future studies should test these outcomes with older adults across a range of health-literacy levels.

The present data support only a limited conclusion: under the tested prompt and configurations, LLM interfaces generated information that experts often rated favorably, while important weaknesses remained. Possible uses such as reinforcing general instructions, explaining terminology, or helping patients prepare questions for clinical appointments are implementation hypotheses rather than outcomes demonstrated by this study. The models should not be used to prescribe exercise intensity, weight-bearing progression, medication changes, or management of suspected complications. Before clinical implementation, prospective studies should evaluate clinician-approved prompts, evidence-based grounding, procedure-specific adaptation, comprehension, trust, behavior, and adverse outcomes in realistic multi-turn interactions. Such studies should also test conflicting or incomplete clinician advice and repeated queries across model updates.

### Strengths and limitations

This study has several strengths. It used a clinically focused question bank covering key domains of older adult hip fracture rehabilitation and compared five widely accessible LLMs using identical questions and a standardized prompt. Responses were independently evaluated by one orthopedic nursing expert and two senior trauma orthopedic physicians, with inter-rater agreement quantified. The use of complementary measures of safety, accuracy, empathy, reliability, information quality, and readability provided a multidimensional assessment. The availability of the question bank, prompt, reference mapping table, and scoring materials also enhances transparency and reproducibility.

Several limitations should be acknowledged. First, each question was submitted only once, so within-model variability, repeated-query reproducibility, and temporal stability were not assessed. Second, the standardized prompt explicitly requested safe, accurate, empathetic, and understandable guidance; the results are therefore prompt-conditioned and cannot be generalized to unguided patient use. Third, only 11 responses across three questions failed the binary safety criterion, precluding a robust inferential comparison among interfaces; the binary endpoint also did not grade the severity, probability, or clinical consequences of potential harm. Fourth, isolated questions rather than realistic multi-turn conversations were evaluated, and patient comprehension, trust, adherence, behavior, and clinical outcomes were not measured. Fifth, model names were removed where feasible, but distinctive formatting, list structures, and linguistic register probably revealed model identity. No formal blinding-success check was performed, so ratings should be regarded as effectively unblinded; empathy and GQS are particularly susceptible to expectancy effects. Sixth, product and model comparability was limited (Table 1): Gemini 2.0 Flash was a lightweight, earlier-generation model, whereas ChatGPT-5.5 and DeepSeek-V4-Pro were the current flagship versions presented in their tested interfaces; ChatGPT-5.5 and Gemini 2.0 Flash were accessed through paid tiers, whereas Microsoft Copilot, DeepSeek-V4-Pro, and Doubao were accessed through free tiers; web search or grounding was enabled for all five; and the underlying model was undisclosed for Copilot and Doubao. Consequently, observed differences may reflect model generation, access tier, retrieval behavior, hidden backend models, interface design, or product configuration rather than model capability alone. Finally, LLM products and deployments evolve rapidly, sometimes without notice. The rankings cannot be generalized beyond the exact interfaces, access tiers, underlying-model disclosures, grounding settings, prompts, and configurations tested during May 1–10, 2026, and should not be treated as durable or current rankings after subsequent updates.

## Conclusion

Within this controlled, prompt-conditioned evaluation, most responses met the prespecified binary safety criterion and received high accuracy ratings, but clinically relevant differences emerged in empathy, reliability, information quality, and readability. This variability is the principal finding and indicates that LLMs are not interchangeable sources of postoperative hip fracture rehabilitation information. The results do not establish that unguided responses are generally safe or suitable for procedure-specific decisions. For older adults and caregivers, LLMs may serve only as supervised adjuncts that reinforce general education while remaining subordinate to individualized surgical and rehabilitation plans. Future studies should evaluate realistic, multi-turn interactions incorporating user characteristics, clinical documents, clinician advice, comprehension, safe application, and clinical usefulness.

## Data Availability

The original contributions presented in the study are included in the article/supplementary material, further inquiries can be directed to the corresponding authors.

## References

[1] Cooper C Campion G Melton LJ. Hip fractures in the elderly: a world-wide projection. Osteoporos Int. (1992) 2:285–9. doi: 10.1007/BF01623184 1421796

[2] Bui M Nijmeijer WS Hegeman JH Witteveen A Groothuis-Oudshoorn CGM. Systematic review and meta-analysis of preoperative predictors for early mortality following hip fracture surgery. Osteoporos Int. (2024) 35:561–74. doi: 10.1007/s00198-023-06942-0 37996546 PMC10957669

[3] Zhang B Dou B Li K. Global, regional, and national burden of hip fractures attributable to falls in older adults: changes from 1990–2021 and 2036 projections. Front Public Health. (2025) 13:1674881. doi: 10.3389/fpubh.2025.1674881 41048275 PMC12488404

[4] O'Connor MI Switzer JA. Management of hip fractures in older adults. J Am Acad Orthop Surg. (2022) 30:e1297–301. doi: 10.5435/JAAOS-D-22-00273 36200818

[5] McDonough CM Harris-Hayes M Kristensen MT Overgaard JA Herring TB Kenny AM . Physical therapy management of older adults with hip fracture. J Orthop Sports Phys Ther. (2021) 51:CPG1–81. doi: 10.2519/jospt.2021.0301 33522384

[6] Zhao L Zhao X Dong B Li X. Effectiveness of home-based exercise for functional rehabilitation in older adults after hip fracture surgery: a systematic review and meta-analysis of randomized controlled trials. PLoS ONE. (2024) 19:e0315707. doi: 10.1371/journal.pone.0315707 39700091 PMC11658508

[7] Armbruster J Bussmann F Rothhaas C Titze N Grützner PA Freischmidt H. “Doctor ChatGPT, can you help me?” the patient's perspective: cross-sectional study. J Med Internet Res. (2024) 26:e58831. doi: 10.2196/58831 39352738 PMC11480680

[8] Gencer B Dogan Ö Çulcu A Ülgen NK Çamoglu C Arslan MM . Internet and social media preferences of orthopaedic patients vary according to factors such as age and education levels. Health Info Libr J. (2024) 41:84–97. doi: 10.1111/hir.12503 37526131

[9] Meng X Yan X Zhang K Liu D Cui X Yang Y . The application of large language models in medicine: a scoping review. iScience. (2024) 27:109713. doi: 10.1016/j.isci.2024.109713 38746668 PMC11091685

[10] Thirunavukarasu AJ Ting DSJ Elangovan K Gutierrez L Tan TF Ting DSW. Large language models in medicine. Nat Med. (2023) 29:1930–40. doi: 10.1038/s41591-023-02448-8 37460753

[11] Singhal K Tu T Gottweis J Sayres R Wulczyn E Amin M . Toward expert-level medical question answering with large language models. Nat Med. (2025) 31:943–50. doi: 10.1038/s41591-024-03423-7 39779926 PMC11922739

[12] Wrenn SP Mika AP Ponce RB Mitchell PM. Evaluating ChatGPT's ability to answer common patient questions regarding hip fracture. J Am Acad Orthop Surg. (2024) 32:656–9. doi: 10.5435/JAAOS-D-23-00877 38743959

[13] Arzu U Gencer B. To self-treat or not to self-treat: evaluating the diagnostic, advisory and referral effectiveness of ChatGPT responses to the most common musculoskeletal disorders. Diagnostics. (2025) 15:1834. doi: 10.3390/diagnostics15141834 40722583 PMC12293552

[14] Gencer B Arzu U Orhan SS Dinçal T Ekinci M. Evaluation of ChatGPT responses about sexual activity after total hip arthroplasty: a comparative study with observers of different experience levels. J Clin Med. (2025) 14:2942. doi: 10.3390/jcm14092942 40363974 PMC12072486

[15] Ong JCL Chang SYH William W Butte AJ Shah NH Chew LST . Ethical and regulatory challenges of large language models in medicine. Lancet Digit Health. (2024) 6:e428–32. doi: 10.1016/S2589-7500(24)00061-X 38658283

[16] Bedi S Liu Y Orr-Ewing L Dash D Koyejo S Callahan A . Testing and evaluation of health care applications of large language models: a systematic review. JAMA. (2025) 333:319–28. doi: 10.1001/jama.2024.21700 39405325 PMC11480901

[17] Tam TYC Sivarajkumar S Kapoor S . A framework for human evaluation of large language models in healthcare derived from literature review. NPJ Digit Med. (2024) 7:258. doi: 10.1038/s41746-024-01258-7 39333376 PMC11437138

[18] Huo B Boyle A Marfo N Tangamornsuksan W Steen JP McKechnie T . Large language models for chatbot health advice studies: a systematic review. JAMA Netw Open. (2025) 8:e2457879. doi: 10.1001/jamanetworkopen.2024.57879 39903463 PMC11795331

[19] The CHART. Collaborative. Reporting guideline for chatbot health advice studies: the CHART statement. JAMA Netw Open. (2025) 8:e2530220. doi: 10.1001/jamanetworkopen.2025.30220 40747871

[20] Rovetta A. Google trends in infodemiology: methodological steps to avoid irreproducible results and invalid conclusions. Int J Med Inform. (2024) 190:105563. doi: 10.1016/j.ijmedinf.2024.105563 39043059

[21] Min K Beom J Kim BR Lee SY Lee GJ Lee JH . Clinical practice guideline for postoperative rehabilitation in older patients with hip fractures. Ann Rehabil Med. (2021) 45:225–59. doi: 10.5535/arm.21110 34233406 PMC8273721

[22] Lin CL Liao CD Lee YH Escorpizo R Liou TH Huang SW. Delphi-based consensus to determine core aspects of post-hip-fracture surgery rehabilitation based on the international classification of functioning, disability, and health. Int J Environ Res Public Health. (2022) 19:15988. doi: 10.3390/ijerph192315988 36498067 PMC9739491

[23] World Medical Association. World Medical Association Declaration of Helsinki: ethical principles for medical research involving human subjects. JAMA. (2013) 310:2191–4. doi: 10.1001/jama.2013.281053 24141714

[24] Ayers JW Poliak A Dredze M Leas EC Zhu Z Kelley JB . Comparing physician and artificial intelligence chatbot responses to patient questions posted to a public social media forum. JAMA Intern Med. (2023) 183:589–96. doi: 10.1001/jamainternmed.2023.1838 37115527 PMC10148230

[25] Charnock D Shepperd S Needham G Gann R. DISCERN: an instrument for judging the quality of written consumer health information on treatment choices. J Epidemiol Community Health. (1999) 53:105–11. doi: 10.1136/jech.53.2.105 10396471 PMC1756830

[26] Moult B Franck LS Brady H. Ensuring quality information for patients: development and preliminary validation of a new instrument to improve the quality of written health care information. Health Expect. (2004) 7:165–75. doi: 10.1111/j.1369-7625.2004.00273.x 15117391 PMC5060233

[27] Silberg WM Lundberg GD Musacchio RA. Assessing, controlling, and assuring the quality of medical information on the internet: Caveant lector et viewor. JAMA. (1997) 277:1244–5. doi: 10.1001/jama.1997.03540390074039 9103351

[28] Bernard A Langille M Hughes S Rose C Leddin D Veldhuyzen van Zanten S. A systematic review of patient inflammatory bowel disease information resources on the World Wide Web. Am J Gastroenterol. (2007) 102:2070–7. doi: 10.1111/j.1572-0241.2007.01325.x 17511753

[29] Lee GG Goodman D Chang TCP. Impact of demographic modifiers on readability of myopia education materials generated by large language models. Clin Ophthalmol. (2024) 18:3591–604. doi: 10.2147/OPTH.S483024 39649984 PMC11625417

[30] Flesch R. A new readability yardstick. J Appl Psychol. (1948) 32:221–33. doi: 10.1037/h0057532 18867058

[31] Coleman M Liau TL. A computer readability formula designed for machine scoring. J Appl Psychol. (1975) 60:283–4. doi: 10.1037/h0076540

[32] Hedman AS. Using the SMOG formula to revise a health-related document. Am J Health Educ. (2008) 39:61–4. doi: 10.1080/19325037.2008.10599016

[33] Koo TK Li MY. A guideline of selecting and reporting intraclass correlation coefficients for reliability research. J Chiropr Med. (2016) 15:155–63. doi: 10.1016/j.jcm.2016.02.012 27330520 PMC4913118

[34] Fleiss JL. Measuring nominal scale agreement among many raters. Psychol Bull. (1971) 76:378–82. doi: 10.1037/h0031619

[35] Friedman M. The use of ranks to avoid the assumption of normality implicit in the analysis of variance. J Am Stat Assoc. (1937) 32:675–701. doi: 10.1080/01621459.1937.10503522

[36] Zhang Y Huang T Liu C Miller AN Yang M Harris IA . Comparative evaluation of large language models for hip fracture-related patient questions: DeepSeek-V3-FW, Gemini 20 Flash, and ChatGPT-45. Digit Health. (2026) 12:20552076251412989. doi: 10.1177/20552076251412989 41623725 PMC12858731

[37] Yau JYS Saadat S Hsu E Murphy LSL Roh JS Suchard J . Accuracy of prospective assessments of 4 large language model chatbot responses to patient questions about emergency care: experimental comparative study. J Med Internet Res. (2024) 26:e60291. doi: 10.2196/60291 39496149 PMC11574488

