# Supplementary material for: Performance of large language models in postoperative hip fracture rehabilitation counseling for older adults: a comparative evaluation of safety, accuracy, reliability, readability, and empathy
Source: Front Public Health. 2026 Sep 30;14:1961597. doi: 10.3389/fpubh.2026.1961597 (PMC13662073; doi:10.3389/fpubh.2026.1961597)
Supplement: Supplementary file 1 [file Table_1.docx]

**Supplementary Material 1 CHART Reporting Checklist**

**Manuscript:** Performance of large language models in postoperative hip fracture rehabilitation counseling for older adults: a comparative evaluation of safety, accuracy, reliability, readability, and empathy

| Section and topic | Item | CHART checklist item | Article page(s) | Section or location in the typeset proof |
| --- | --- | --- | --- | --- |
| Title and abstract | 1a | State that the study assesses generative AI-driven chatbots for clinical evidence or health advice. | p. 1 | Title; Abstract - Objective and Methods |
| Title and abstract | 1b | Apply a structured abstract format, if applicable. | pp. 1-2 | Abstract - Objective, Methods, Results, and Conclusions |
| Introduction | 2a | State the scientific background, rationale, and healthcare context. | pp. 2-3 | Introduction |
| Introduction | 2b | State the aims and research questions, including target audience, intervention, comparators, and outcomes. | p. 3 | Introduction - final paragraph (study hypothesis and null hypothesis) |
| Methods - Model identifiers | 3a | State the model/chatbot name, version identifiers, and release or last-update dates. | pp. 3-5 | Methods - Generative AI chatbots, model identifiers, and access; Table 1 |
| Methods - Model identifiers | 3b | State whether models/chatbots are open-source or closed-source/proprietary. | pp. 3-5 | Methods - Generative AI chatbots, model identifiers, and access; Table 1 |
| Methods - Model details | 4a | State whether the model was a base model, novel base model, tuned model, or fine-tuned model. | p. 5 | Table 1 - Tested identifier and product/model status |
| Methods - Model details | 4b | If a base model is used, cite its development sufficiently to identify the model. | pp. 3-5 | Methods - Generative AI chatbots, model identifiers, and access; Table 1 - Underlying-model disclosure/comparability |
| Methods - Model details | 4c | For novel base, tuned, or fine-tuned models, describe deployment data and parameters. | pp. 3-5 | Not applicable - publicly accessible interfaces were evaluated; no model was developed, trained, or fine-tuned by the study team |
| Methods - Prompt engineering | 5a | Describe the evolution of prompt development. | p. 5 | Methods - Prompt engineering and query strategy |
| Methods - Prompt engineering | 5ai | Describe the sources of prompts. | p. 5 | Methods - Prompt engineering and query strategy |
| Methods - Prompt engineering | 5aii | State the number and characteristics of individuals involved in prompt engineering. | p. 5 | Methods - Prompt engineering and query strategy |
| Methods - Prompt engineering | 5aiii | Provide details of patient/public involvement during prompt engineering. | p. 5 | Methods - Prompt engineering and query strategy |
| Methods - Prompt engineering | 5b | Provide the study prompts. | p. 5 | Standardized prompt used for each query |
| Methods - Query strategy | 6a | State the route of access to the generative AI model. | pp. 3-5 | Methods - Generative AI chatbots, model identifiers, and access; Table 1 |
| Methods - Query strategy | 6b | State the dates and locations of queries, including day/month/year and city/country. | p. 5 | Table 1 footnote and accompanying Methods paragraph (China; May 1-10, 2026) |
| Methods - Query strategy | 6c | Describe whether prompts were entered in separate chat sessions. | p. 5 | Methods - Prompt engineering and query strategy |
| Methods - Query strategy | 6d | Provide all chatbot outputs/responses. | pp. 5, 12 | Methods - Prompt engineering and query strategy; Data availability statement; Supplementary material |
| Methods - Performance evaluation | 7a | Define the ground truth or reference standard for successful performance. | pp. 3, 6 | Question bank development; Evaluation indicators - Accuracy, safety, and empathy |
| Methods - Performance evaluation | 7b | Describe the performance evaluation process. | pp. 5-7 | Response collection and data management; Evaluation indicators; Rater evaluation; Statistical analysis |
| Methods - Performance evaluation | 7bi | State the number and characteristics of team members involved in evaluation. | p. 6 | Rater evaluation |
| Methods - Performance evaluation | 7bii | Provide details of patient/public involvement during evaluation. | pp. 3, 6 | Ethics statement; Rater evaluation |
| Methods - Performance evaluation | 7biii | State whether evaluators were blinded to chatbot identity. | pp. 5-6 | Response collection and data management; Rater evaluation |
| Methods - Sample size | 8 | Report how the sample size was determined. | pp. 6-7 | Sample size |
| Methods - Data analysis | 9a | Describe statistical methods, including reproducibility evaluation. | pp. 5, 7 | Prompt engineering and query strategy (repeated-query reproducibility); Statistical analysis |
| Methods - Data analysis | 9ai | Report the measures used for performance evaluation. | pp. 6-7 | Evaluation indicators; Statistical analysis |
| Results | 10a | Report performance evaluation and alignment with the ground truth/reference standard. | pp. 7-10 | Results - Question bank and response dataset; Inter-rater agreement; Safety, accuracy, and empathy; Reliability and quality indicators; Readability analyses |
| Results | 10b | For deviations from the ground truth/reference standard, state the nature of the differences. | pp. 7-8 | Results - Safety, accuracy, and empathy; Table 2; Supplementary Material 8 |
| Results | 10c | Report potentially harmful, biased, or misleading responses. | pp. 7-8 | Results - Safety, accuracy, and empathy; Table 2; Supplementary Material 8 |
| Discussion | 11a | Interpret the findings in the context of relevant evidence. | pp. 9-11 | Discussion |
| Discussion | 11b | Describe strengths and limitations. | p. 11 | Strengths and limitations |
| Discussion | 11c | Describe implications for practice, education, policy, regulation, and research. | pp. 10-11 | Discussion; Conclusion |
| Open science - Disclosures | 12a | Report conflicts of interest. | p. 12 | Conflict of interest |
| Open science - Funding | 12b | Report funding sources and their role. | p. 12 | Funding |
| Open science - Ethics | 12c | Describe the ethical approval process. | pp. 3, 12 | Ethics statement |
| Open science - Ethics | 12ci | Describe safeguards for patient health information privacy. | pp. 3, 12 | Ethics statement |
| Open science - Ethics | 12cii | State whether permission/licensing was obtained for original copyrighted data. | pp. 3, 12 | Ethics statement - no usernames, identifiable quotations, private posts, or protected health information were retained |
| Open science - Protocol | 12d | Provide a study protocol. | p. 3 | Study design and reporting framework; no separate protocol or registration is reported in the typeset proof |
| Open science - Data availability | 12e | State where data, the code repository, and model parameters can be accessed. | pp. 5, 12 | Generative AI chatbots, model identifiers, and access; Prompt engineering and query strategy; Data availability statement |

**Abbreviations:** AI, artificial intelligence; LLM, large language model.
