# Supplementary material for: Performance of large language models in postoperative hip fracture rehabilitation counseling for older adults: a comparative evaluation of safety, accuracy, reliability, readability, and empathy
Source: Front Public Health. 2026 Sep 30;14:1961597. doi: 10.3389/fpubh.2026.1961597 (PMC13662073; doi:10.3389/fpubh.2026.1961597)
Supplement: Supplementary file 4 [file Table_5.docx]

The readability of the responses was assessed using six widely recognized readability metrics, using online calculator (https://readabilityformulas.com).

AStatistical analysisd Readability Index (ARI) : 4.71 * ( characters / words)) + (0.5 * (words / sentences)) - 21.43

Flesch Reading Ease Score (FRES) : 206.835 - (1.015 * (words / sentences)) - (84.6 * syllables / words)

Gunning Fog Index ( GFI ) : 0.4 * ( words / ( sentences + compound sentences)) + 100 * (FOG hard words / words)

Flesch-Kincaid Grade Level (FKGL) : 0.39( words /sentences ) + 11.8( syllables /words ) − 15.59

Coleman - Liau Readability Index (CLI) : 5.88 * (letters / words) - 0.296 * (sentences / words) - 15.8

The SMOG Index(SMOG): 1.043 * Sqrt(hard words * (30 / sentences)) + 3.1291
