# Supplementary material for: Performance of large language models in postoperative hip fracture rehabilitation counseling for older adults: a comparative evaluation of safety, accuracy, reliability, readability, and empathy
Source: Front Public Health. 2026 Sep 30;14:1961597. doi: 10.3389/fpubh.2026.1961597 (PMC13662073; doi:10.3389/fpubh.2026.1961597)
Supplement: Supplementary file 5 [file Table_6.docx]

## Table S1. Inter-rater agreement

| **Metric** | **Agreement index** | **Estimate** | **95% CI** | **P value** | **Complete items** | **No. of raters** | **Note** |
| --- | --- | --- | --- | --- | --- | --- | --- |
| Safety | Fleiss' kappa | 0.855 | 0.704-0.970 | <0.001 | 210 | 3 | Primary agreement index for Safety; 95% CI based on 3000 nonparametric bootstrap resamples. A negative lower CI may occur when unsafe outcomes are extremely sparse because kappa can theoretically be below 0; this does not by itself indicate a coding error. |
| Accuracy | ICC(2,1) | 0.868 | 0.838-0.894 | <0.001 | 210 | 3 | Two-way random-effects, absolute-agreement, single-measure ICC. |
| Empathy | ICC(2,1) | 0.829 | 0.791-0.862 | <0.001 | 210 | 3 | Two-way random-effects, absolute-agreement, single-measure ICC. |
| DISCERN | ICC(2,1) | 0.878 | 0.849-0.902 | <0.001 | 210 | 3 | Two-way random-effects, absolute-agreement, single-measure ICC. |
| EQIP | ICC(2,1) | 0.884 | 0.857-0.907 | <0.001 | 210 | 3 | Two-way random-effects, absolute-agreement, single-measure ICC. |
| JAMA | ICC(2,1) | 0.854 | 0.820-0.882 | <0.001 | 210 | 3 | Two-way random-effects, absolute-agreement, single-measure ICC. |
| GQS | ICC(2,1) | 0.841 | 0.805-0.872 | <0.001 | 210 | 3 | Two-way random-effects, absolute-agreement, single-measure ICC. |

Note: Safety was assessed using Fleiss' kappa. Accuracy, Empathy, DISCERN, EQIP, JAMA and GQS were assessed using ICC(2,1).

Table S2. Pairwise comparisons of safety

| **Comparison** | **N pairs** | **Safe rate difference, %** | **Safe to unsafe, n** | **Unsafe to safe, n** | **Adjusted P** | **Note** |
| --- | --- | --- | --- | --- | --- | --- |
| Copilot vs DeepSeek | 42 | 2.38 | 1 | 0 | 1.000 | Pairwise McNemar test |
| Copilot vs Doubao | 42 | -2.38 | 0 | 1 | 1.000 | Pairwise McNemar test |
| Copilot vs ChatGPT | 42 | 2.38 | 1 | 0 | 1.000 | Pairwise McNemar test |
| Copilot vs Gemini | 42 | 0.00 | 1 | 1 | 1.000 | Pairwise McNemar test |
| DeepSeek vs Doubao | 42 | -4.76 | 0 | 2 | 1.000 | Pairwise McNemar test |
| DeepSeek vs ChatGPT | 42 | 0.00 | 0 | 0 |  | No discordant pairs; McNemar test not applicable |
| DeepSeek vs Gemini | 42 | -2.38 | 0 | 1 | 1.000 | Pairwise McNemar test |
| Doubao vs ChatGPT | 42 | 4.76 | 2 | 0 | 1.000 | Pairwise McNemar test |
| Doubao vs Gemini | 42 | 2.38 | 1 | 0 | 1.000 | Pairwise McNemar test |
| ChatGPT vs Gemini | 42 | -2.38 | 0 | 1 | 1.000 | Pairwise McNemar test |

Note: Pairwise comparisons were performed using McNemar tests with Benjamini-Hochberg correction.

## Table S3. Significant pairwise comparisons for non-binary outcomes

| **Metric** | **Comparison** | **N pairs** | **Mean difference** | **Median difference** | **Adjusted P** |
| --- | --- | --- | --- | --- | --- |
| Empathy | Copilot vs Doubao | 42 | -0.22 | 0.00 | 0.047 |
| Empathy | Copilot vs Gemini | 42 | -0.34 | -0.15 | 0.004 |
| Empathy | DeepSeek vs Gemini | 42 | -0.23 | 0.00 | 0.046 |
| Empathy | ChatGPT vs Gemini | 42 | -0.31 | 0.00 | 0.004 |
| DISCERN | Copilot vs DeepSeek | 42 | -3.80 | -3.70 | <0.001 |
| DISCERN | Copilot vs Doubao | 42 | -3.77 | -4.15 | <0.001 |
| DISCERN | Copilot vs ChatGPT | 42 | -1.86 | -1.85 | 0.004 |
| DISCERN | Copilot vs Gemini | 42 | -4.74 | -4.85 | <0.001 |
| DISCERN | DeepSeek vs ChatGPT | 42 | 1.94 | 1.00 | 0.012 |
| DISCERN | Doubao vs ChatGPT | 42 | 1.90 | 1.70 | 0.007 |
| DISCERN | Doubao vs Gemini | 42 | -0.97 | -1.00 | 0.027 |
| DISCERN | ChatGPT vs Gemini | 42 | -2.87 | -2.00 | <0.001 |
| EQIP | Copilot vs DeepSeek | 42 | -3.17 | -4.15 | <0.001 |
| EQIP | Copilot vs Doubao | 42 | -3.41 | -4.15 | <0.001 |
| EQIP | Copilot vs Gemini | 42 | -3.96 | -5.00 | <0.001 |
| EQIP | DeepSeek vs ChatGPT | 42 | 2.10 | 0.80 | 0.019 |
| EQIP | DeepSeek vs Gemini | 42 | -0.79 | 0.00 | 0.026 |
| EQIP | Doubao vs ChatGPT | 42 | 2.34 | 1.70 | 0.002 |
| EQIP | ChatGPT vs Gemini | 42 | -2.90 | -2.50 | <0.001 |
| GQS | Copilot vs DeepSeek | 42 | -0.41 | 0.00 | 0.005 |
| GQS | Copilot vs Doubao | 42 | -0.33 | 0.00 | 0.003 |
| GQS | Copilot vs Gemini | 42 | -0.44 | 0.00 | 0.003 |
| GQS | ChatGPT vs Gemini | 42 | -0.20 | 0.00 | 0.021 |
| ARI | Copilot vs Doubao | 42 | -2.26 | -2.60 | 0.003 |
| CL | Copilot vs ChatGPT | 42 | -1.35 | -1.32 | 0.023 |
| CL | Copilot vs Gemini | 42 | -1.39 | -1.32 | 0.003 |
| CL | DeepSeek vs ChatGPT | 42 | -0.88 | -1.08 | 0.028 |
| CL | DeepSeek vs Gemini | 42 | -0.92 | -0.85 | 0.003 |
| FKGL | Copilot vs Doubao | 42 | -2.56 | -2.48 | 0.003 |
| FKGL | Copilot vs ChatGPT | 42 | -1.61 | -2.65 | 0.048 |
| FKGL | Doubao vs Gemini | 42 | 1.56 | 1.02 | 0.048 |
| GFI | DeepSeek vs ChatGPT | 42 | -1.35 | -1.15 | 0.044 |
| SMOG | Copilot vs Doubao | 42 | -0.99 | -1.43 | 0.045 |

Note: Pairwise comparisons were performed using paired Wilcoxon signed-rank tests with Benjamini-Hochberg correction. Only significant comparisons are shown.
